# Biomarker-guided antibiotic stewardship in neonatology: time for implementation?

**DOI:** 10.1038/s41390-025-03807-6

**Published:** 2025-01-14

**Authors:** Lizelle Van Wyk, Lizel G. Lloyd

**Affiliations:** Dept of Paediatrics and Child Health, Stellenbosch University, Cape Town, South Africa

Neonatal sepsis continues to be a leading cause of global neonatal mortality. Despite advances in knowledge and technology, neonatal sepsis lacks a universally accepted and precise definition, depending on non-specific clinical signs, supported by diverse laboratory tests, performed at varying timepoints and utilizing a variety of cut-off values.^1^ Consequently, antibiotics are frequently overprescribed for presumed sepsis, contributing to the global antibiotic resistance health crisis.^2^

The diagnostic gold standard for neonatal sepsis remains a positive blood culture. However, this is dependent on proper aseptic technique, sufficient blood sample volume, and may take up to 72 h for a positive result, often leading to unnecessary antibiotic exposure.^3^ Various biomarkers have been used to support a diagnosis of sepsis, with the most common being C-reactive protein and procalcitonin (PCT).^4^

Point-of-care (POC) tests are able to provide extra-laboratory, bedside testing, do not require specialized medical personnel, and are able to provide rapid results.^5^ Faster turn-around times of POC infective biomarker results may lead to rapid diagnosis of sepsis and initiation of treatment but also, possibly, to the exclusion of infection and cessation of unnecessary antibiotics, allowing improved antibiotic stewardship.^4^ POC tests also hold promise as sepsis screening tools in lower-middle-income countries, where sepsis and sepsis-related mortality is high, and testing ability, related to resource and cost restrictions, is poor.

In this edition of *Pediatric Research*, Armstrong et al.^6^, performed a prospective observational study in neonates with late onset sepsis (LOS) showing the ability of a PCT < 0.5 μg/L to rule out blood stream infections (BSI) and Bell’s grade III necrotizing enterocolitis (NEC) with a high sensitivity and specificity but moderate sensitivity and specificity for ruling out ventilator-associated pneumonia (VAP). The authors also showed the continued difference in PCT levels between BSI and non-BSI, as well as NEC grade III and non-NEC groups at 24 and 72 h, suggesting the possibility of earlier cessation of antibiotics.

Armstrong et al. demonstrated the ability to discriminate between blood culture negative and BSI episodes using PCT > 0.5 μg/L performed at 24 and 72 h after clinical suspicion of sepsis. Literature, however, is ambivalent regarding the ability of PCT to discriminate between clinically suspected, culture-negative, and culture-positive sepsis.^7^ However, variable sepsis definitions, different study populations, variable time-points of biomarker testing, and intended outcomes of studies,^1^ make the decision to use biomarker-guided discriminative diagnosis difficult.

Prediction of NEC is the long-held aspiration of many a neonatologist, with the differentiation of NEC from non-NEC entities being difficult, especially in non-surgical grades.^8^ Armstrong et al., demonstrated that PCT was able to discriminate between feeding intolerance and Bell’s grade III NEC. Despite the relatively small number of NEC cases and the inclusion of high grade-NEC, this result remains significant as it may support cessation of antibiotics based on low PCT values in stable infants.

Despite their findings supporting early cessation of antibiotics, Armstrong et al., indicate that nearly 50% of infants continued to receive antibiotics. This may be attributed to the lack of a consensus definition for neonatal sepsis, distrust of the accuracy of biomarkers, confusion about the interpretation of a negative blood culture due to paucicellular sampling, and “erring on the side of caution” by clinicians, despite evidence to support the absence of sepsis.^7,9^ Improving biomarker accuracy, continued education of clinicians, and incorporating biomarkers into clinical decision support systems, may help address these challenges.

POC testing allows for the use of minimal blood volume and a shorter turn-around time to result availability. As shown by Armstrong et al., the blood volume required for this POC PCT test was 20 μL, significantly less than the standard volume of blood needed (250–500 μl) for formal laboratory testing. This may contribute to less blood loss in very low birth weight infants, decreasing the need for red blood cell transfusions. In Armstrong et al.’s study, POC-PCT results were available within 20 min, similar to a diagnostic accuracy study, where POC neonatal sepsis biomarkers were available in less than 15 min as compared to a mean laboratory reporting time of 366 min.^10^ This may enhance clinical decision-making and enable early cessation of antibiotics, especially in infants who are clinically stable or where significant sepsis is not suspected.

PCT remains the most common biomarker used in antibiotic stewardship programs.^11^ Consensus statements, in this regard, have been published in high^12^ and lower-middle-income countries for use in adults.^13,14^ Few studies have been performed in pediatrics, but would also seem to indicate improved antibiotic stewardship with PCT-guided antibiotic use.^15,16^ PCT-guided antibiotic stewardship programs have shown significant reductions in antibiotic use and exposure, with no increase in mortality or re-infection.^11^ Armstrong et al. also suggest that using a PCT < 0.5 μg/L at 24 h could reduce antibiotic exposure by 30%. Decreased antibiotic use may positively affect micro-organism resistances rates as well as lead to hospital cost reductions. Antibiotic stewardship in neonates could, therefore, potentially be enhanced with the use of POC PCT testing.

Neonatal sepsis remains a significant cause of mortality and morbidity. Biomarkers, such as PCT, are commonly used to rule out and monitor the progress of sepsis. Clinicians are often concerned about the lack of discriminatory ability of these biomarkers. PCT-guided clinical decision support algorithms may help address these worries, by providing more accurate and timely guidance for antibiotic management. Although biomarkers may be used as rule-out tests for sepsis and contribute to antibiotic stewardship, multiple other organizational aspects (test validation, timing and frequency of testing, guideline development, resource allocation) need to be considered prior to implementing a biomarker-only algorithm for antibiotic stewardship. Although PCT-guided antibiotic stewardship programs show promise, more research and evaluation are needed before implementation into routine clinical practice.
